# Azilsartan protects against hyperglycemia-induced hyperpermeability of the blood-brain barrier

**DOI:** 10.1080/21655979.2021.1948950

**Published:** 2021-07-16

**Authors:** Jing Han, Hua Tang, Longfei Yao, Erliang Jin, Wanxi Pan, Shaojun Chen

**Affiliations:** Department of Neurosurgery, the People’s Hospital of China Three Gorges University, Yichang, Hubei, China

**Keywords:** Azilsartan, diabetes mellitus, blood-brain barrier, KLF2

## Abstract

Diabetes mellitus (DM) is a complex metabolic disease with significant neurological complications and is reported to be closely related to the blood-brain barrier (BBB) disruption. Azilsartan is an antagonist of the Angiotensin II receptor developed for the treatment of hypertension, and it has been recently reported to have neuroprotective effects. The present study aims to investigate the protective effect of Azilsartan against hyperglycemia-induced BBB disruption and its underlying mechanism. Male db/db mice were treated with Azilsartan (20 μg/day) for 10 consecutive days. Compared to the control group, increased BBB permeability, suppressed occludin expression, excessive release of inflammatory factors, and downregulation of krüppel-like factor 2 (KLF2) were observed in diabetic mice, all of which were dramatically reversed by Azilsartan treatment. In the *in vitro* experiments, elevated endothelial permeability and decreased expression of occludin and KLF2 were observed in high glucose-challenged endothelial cells, which were significantly alleviated by Azilsartan. Lastly, the silencing of KLF2 abolished the protective effects of Azilsartan against the high glucose-induced expression of occludin and endothelial monolayer permeability in bEnd.3 brain endothelial cells. Based on these observations, we concluded that Azilsartan protected against hyperglycemia-induced hyperpermeability of BBB via the KLF2/occludin axis.

## Graphical abstract

**Figure:**
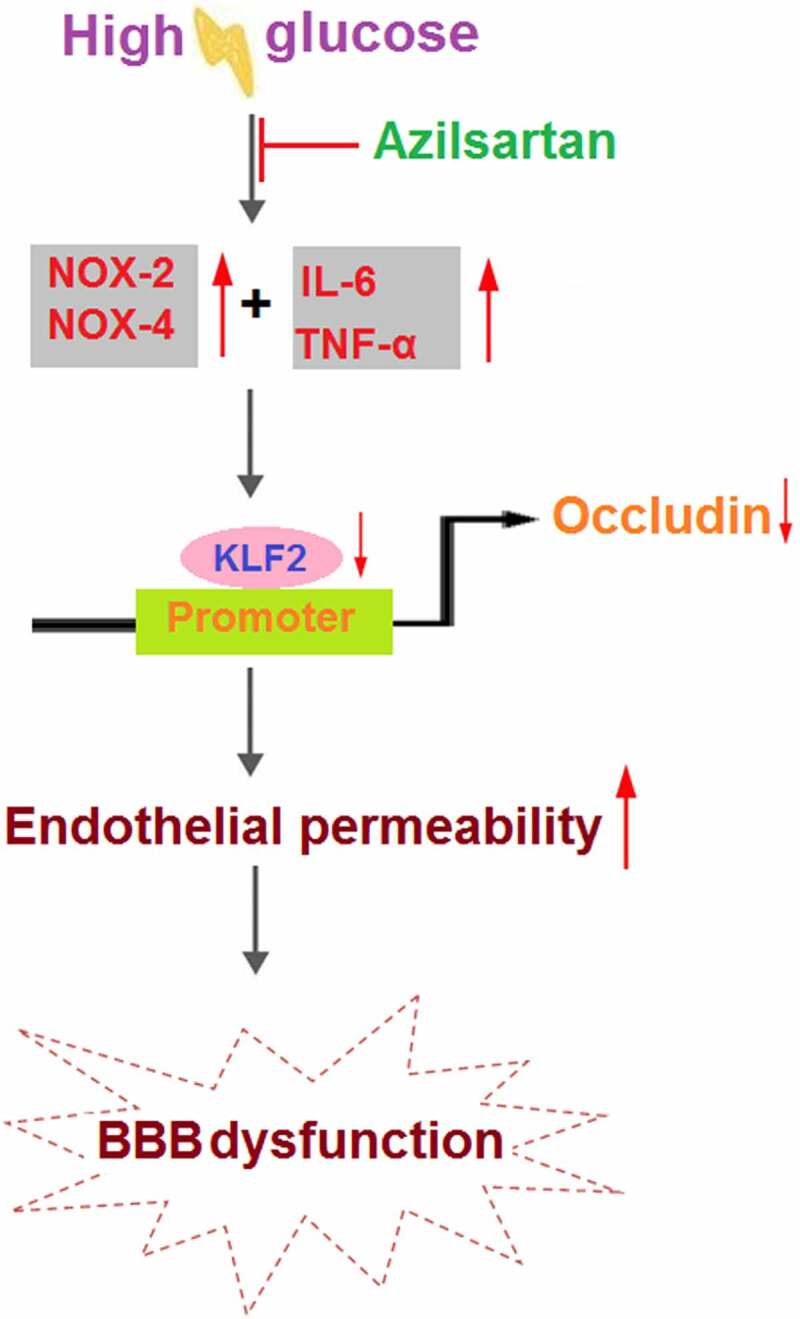

## Introduction

Diabetes mellitus (DM) is a common metabolic disease and is divided into type I and type II diabetes mellitus based on the dependence on the intake of exogenous insulin by DM patients, among which type II diabetes mellitus (T2DM) occupies approximately 90% in the total population. According to the statistics of the International Diabetes Foundation in 2011, approximately 90 million patients were diagnosed with T2DM and it is predicted that 129.7 million patients will be diagnosed with T2DM by 2030, occupying 12.1% of the total population [1]. As a systemic disease, T2DM can induce a series of complications, including diabetic eye disease, kidney disease, cardiovascular disease, encephalopathy, etc. Among them, neurological disorders induced by T2DM have attracted much attention in recent years [2]. According to statistics, about 60%-70% of T2DM patients are diagnosed with mild to moderate cognitive impairment the main characteristics of which are impaired memory, poor thinking ability, impaired motor coordination, and mood disorders [3,4]. It is reported that the risk of Alzheimer’s disease diagnosed in T2DM patients is 2-3-fold than that in healthy people [5]. One of the main inducements of neurological disorders triggered by T2DM is the disruption of the blood-brain barrier (BBB). As the development of T2DM, the lesion on the brain microvascular will be aggravated, which further contributes to the disruption of the physiological and structure of BBB [6]. The BBB is mainly composed of brain microvascular endothelial cells, tight junctions between endothelial cells, basal membrane, and the glial membrane outside capillaries [7]. Chehade reported that the expression of occludin, a tight junction protein located in the BBB, is suppressed significantly in diabetic rats [8]. VanGilder claimed that the increased BBB permeability and downregulated occludin were observed in diabetic rats [9]. Moreover, the interaction between Aβ and the receptor for advanced glycation end-product (RAGE) suppressed the expression of occludin and elevated the expression of matrix metalloproteinases (MMPs) by mediating the Ca^2+^- neurocalcin signaling pathway, further damaging the tight junction in the BBB, altered the morphology of brain microvascular, and aggravated the accumulation of Aβ in the brain, indicating that the decreased expression of tight junction proteins induced by T2DM might be responsible for the neurological disorders [10]. Chiplunkar [11] pointed out that the expression level of the tight junction proteins, such as occludin, could be regulated by the Krüppel-like factor 2 (KLF2). Therefore, targeting KLF2/occludin axis mediated brain endothelial permeability might be an effective method for the treatment of T2DM-induced neurological disorders.

**Figure 1.**
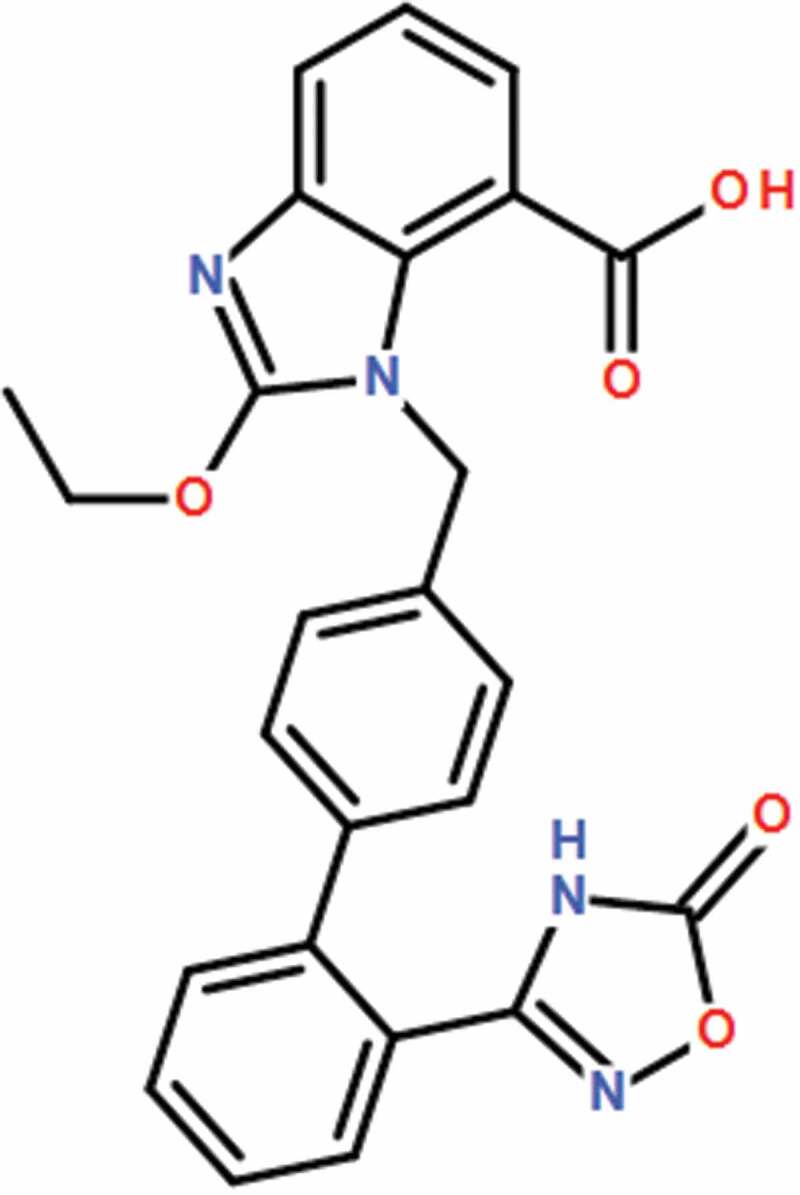
Molecular structure of Azilsartan

Azilsartan (Figure 1) is a new generation of antagonists of the Angiotensin II receptor developed for the treatment of clinical hypertension. It suppresses vasoconstriction by specifically targeting the Angiotensin II receptor to competitively antagonize the function of Angiotensin II [12,13]. Recently, the significant neuroprotective effects of Azilsartan have been widely reported [14,15]. However, it is unknown whether Azilsartan possesses an efficacy against BBB impairment in diabetes. In the present study, the protective effect of Azilsartan on hyperglycemia-induced hyperpermeability of the BBB will be investigated in both *in vivo* and *in vitro* to explore the potential therapeutic properties of Azilsartan against T2DM-induced neurological disorders.

## Materials and methods

### Animal experiments

Male leptin receptor-deficient mice (db/db) and control mice (db/+) were purchased from Beijing Vital River Laboratory Animal Technology Co. Ltd at the ages of 16 weeks. Azilsartan (20 μg/day) was administered for 10 consecutive days, with normal saline administration taken as a negative control. Experimental protocols were approved by the Animal Experiments Ethics Committee of the People’s Hospital of China Three Gorges University.

## Cell culture and treatments

The bEnd.3 mouse brain endothelial cells were obtained from American Type Culture Collection (Manassas, VA, USA) and cultured in the DMEM medium supplemented with 10% fetal bovine serum (FBS), 100 units/mL of penicillin, and 100 μg/mL of streptomycin at 37℃ and 5% CO_2_.

## Measurement of the BBB permeability with Evan’s blue

20 hours after the last administration of Azilsartan, the animals were injected with 2% Evans blue (Sigma, Missouri, USA) dissolved in sterile saline through the tail vein (4 mL/kg), and then circulated for 4-hours. After being transcardially perfused with saline and euthanasia, the brain tissues of the animals were removed and homogenized in 2 mL dimethylformamide (DMF), followed by incubation at 55℃ for 18 hours. Subsequently, the homogenates were centrifugated at 10,000 g for 20 minutes. Lastly, the absorbance of the samples at 620 nm was detected with an EnSpire microplate reader (PerkinElmer, Massachusetts, USA) [16].

## Isolation of brain vessels

The brain cortex isolated from animals was minced and homogenized in the buffer 1 (8 g L^−1^ NaCl, 400 mg L^−1^ KCl, 185.4 mg L^−1^ CaCl_2_.2H_2_O, 60 mg L^−1^ KH_2_PO_4_, 200 mg L^−1^ MgSO_4_ · 7H_2_O, 350 mg L^−1^ NaHCO_3_, 1 g L^−1^ D-glucose, 90 mg L^−1^ Na_2_HPO_4_.7H_2_O, and 2.4 mg L^−1^ HEPES), followed by adding the protease inhibitor and centrifugation at 2000 g for 10 minutes at 4℃. Subsequently, the pellet was resuspended in 16% dextran, followed by centrifugation at 5500 g for 15 minutes. Then, the supernatant was transferred to a new tube and centrifugated at 5500 g for another 15 minutes, followed by resuspending the pellets using 10 mL buffer 2 (buffer 1 supplemented with 5 mg mL^−1^ bovine serum albumin). After filtration using a 100 µm nylon mesh, the remaining samples were passed through a 20 µm nylon mesh and the retrained fraction was moved to microcentrifuge tubes, followed by centrifugation at 1000 g for 5 minutes at 4℃. Lastly, the resultant microvessel pellet was collected in 1 mL isotonic buffer for subsequent experiments.

## Immunostaining assay

The brain cortex isolated from animals was fixed in 2% formaldehyde solution and embedded in paraffin, followed by being cut into 4-μm-thick sections. The sections were frozen and placed on glass slides, followed by blocking with 5% BSA solution. After washing with PBS buffer, the slides were incubated with the primary antibody against occludin (1:200, CST, Boston, USA) at room temperature for 2 hours. After washing with PBS buffer, the slides were incubated with the secondary antibodies conjugated with TRITC. The slices were mounted with DAPI. The samples, without incubating with the primary antibody, were taken as the negative control. Finally, the fluorescence microscope (Olympus, Toyko, Japan) was used to take images of the fluorescence in each group.

## Real-time PCR analysis

The SuperScript III First-Strand Synthesis Kit (Invitrogen, California, USA) was used to transcribe the isolated total RNAs into cDNA, and further amplified with the SYBR GreenER qPCR Supermix on an ABI PRISM 7900 HT (Applied Biosystems, California, USA). Subsequently, the crossing points were determined with the light cycler analysis software using the second derivative method, followed by calculating the efficiency with the relative standard curve method. Data were normalized to GAPDH and the relative expression level of target genes was determined using the 2^−ΔΔt^ method [17].

## ELISA assay

The concentrations of IL-6 and TNF-α in brain vessels of diabetic mice were evaluated with ELISA assay. In brief, after centrifugation at 1000 g for 10 minutes, the supernatant was collected and moved to a 96-well plate, followed by incubating with 1% BSA for 1 hour to remove the nonspecific binding proteins. Subsequently, the samples were added with the primary antibodies at room temperature for 1 hour, followed by incubating streptavidin-horseradish peroxidase (HRP) conjugated secondary antibodies at room temperature for 20 minutes. The substrate was then added. Finally, the absorbance at 450 nm was measured using the microplate spectrophotometer (Thermo, Massachusetts, USA).

## Western blot analysis

The brain vessels or bEnd.3 brain endothelial cells were lysed using the lysis buffer (Invitrogen, California, USA) followed by extracting the total proteins for quantification. Approximately 40 μg proteins were loaded and separated with SDS-PAGE [18], and further transferred to the PVDF membrane (Invitrogen, California, USA), followed by incubating with 5% BSA to remove the nonspecific binding proteins. Subsequently, the membrane was incubated with the primary antibody against occludin (1:1000, R&D systems, USA), KLF2 (1:1000, R&D systems, USA), and β-actin (1:1000, R&D systems, USA) at 4℃ overnight. Membranes were then incubated with the secondary antibody at room temperature for 1.5 hours, followed by incubation with ECL solution. Lastly, the bands were exposed to the Tonan exposure (Tonan, Shanghai, China), followed by quantifying the relative expression of proteins using the Image J software.

## Endothelial monolayer permeability with fluorescein isothiocyanate (FITC)-dextran permeation

The treated bEnd.3 brain endothelial cells were planted in a 24-well plate and the transwell inserts were rinsed with Hank’s balanced salt solution (HBSS), followed by being placed on a 24-well plate containing 600 μL of HBSS each well. Subsequently, the upper chamber was added with 200 μL of 1 mg/mL FITC-dextran (Sigma-Aldrich, USA), followed by incubation at 37 °C for 15 minutes. Lastly, the intensity of the dye in the lower chamber was spectrophotometrically assessed in a clear bottom black-walled microplate at 485 nm excitation and 520 nm emission.

## Data analysis

Data are presented as mean ± standard deviation S.D. Significance was determined by analysis of variance (ANOVA) followed by Tukey’s posthoc test for multiple comparisons. p < 0.05 was considered significant.

## Results

In this study, we used a leptin receptor-deficient (db/db) mouse model, to investigate the effects of Azilsartan on diabetes-associated BBB permeability and the expression of tight junction protein occludin. In the *in vitro* experiments, we reported that Azilsartan ameliorated high glucose-induced endothelial hyperpermeability mediated by the KLF2/occludin axis.

**Azilsartan mitigated diabetes-induced BBB permeability and preserved the expression of the tight junction protein occludin**

**Figure 2.**
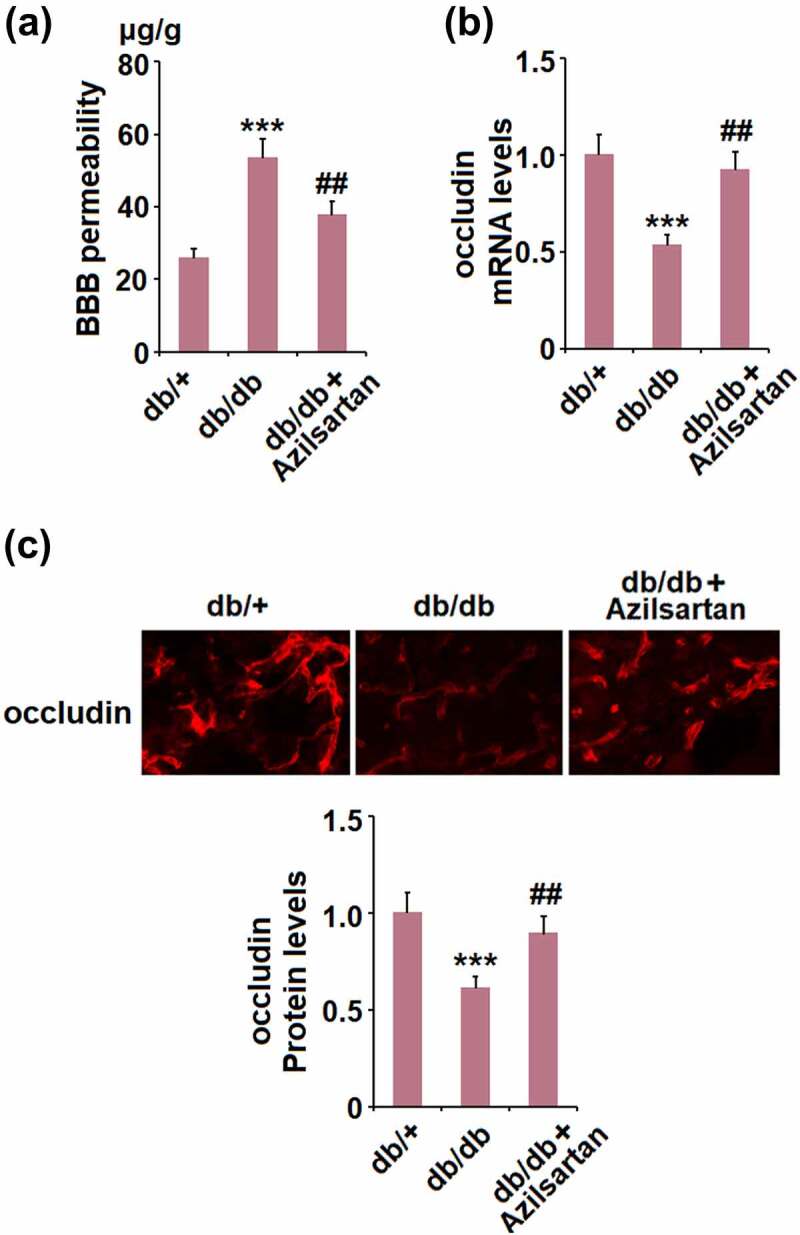
Azilsartan mitigates diabetes-induced BBB permeability and preserves the expression of the tight junction protein occludin. (A). BBB permeability was measured with Evan’s blue; (B). mRNA levels of occludin in isolated brain vessels; (C). Protein levels of occludin were examined in the cortex of mice brains using immunostaining (***, p < 0.005 vs. db/+ group; ##, P < 0.01 vs. db/db group)

First, we evaluated the protective effects of Azilsartan on BBB permeability in diabetic mice. As shown in Figure 2a, compared to the control, the BBB permeability was significantly elevated from 25.6 μg/g tissue to 53.2 μg/g tissue in T2DM mice, but dramatically suppressed to 37.5 μg/g tissue by treatment with Azilsartan. In addition, the expression of the tight junction protein occludin in isolated vessels of T2DM mice was significantly lower than that of normal mice and was greatly elevated by the administration of Azilsartan (Figures 2b-c). These data indicate that the impaired BBB permeability and downregulated tight junction protein occludin in T2DM mice were significantly alleviated by Azilsartan.

**Azilsartan suppressed the expressions of the pro-inflammatory cytokines in brain vessels of diabetes mice**

**Figure 3.**
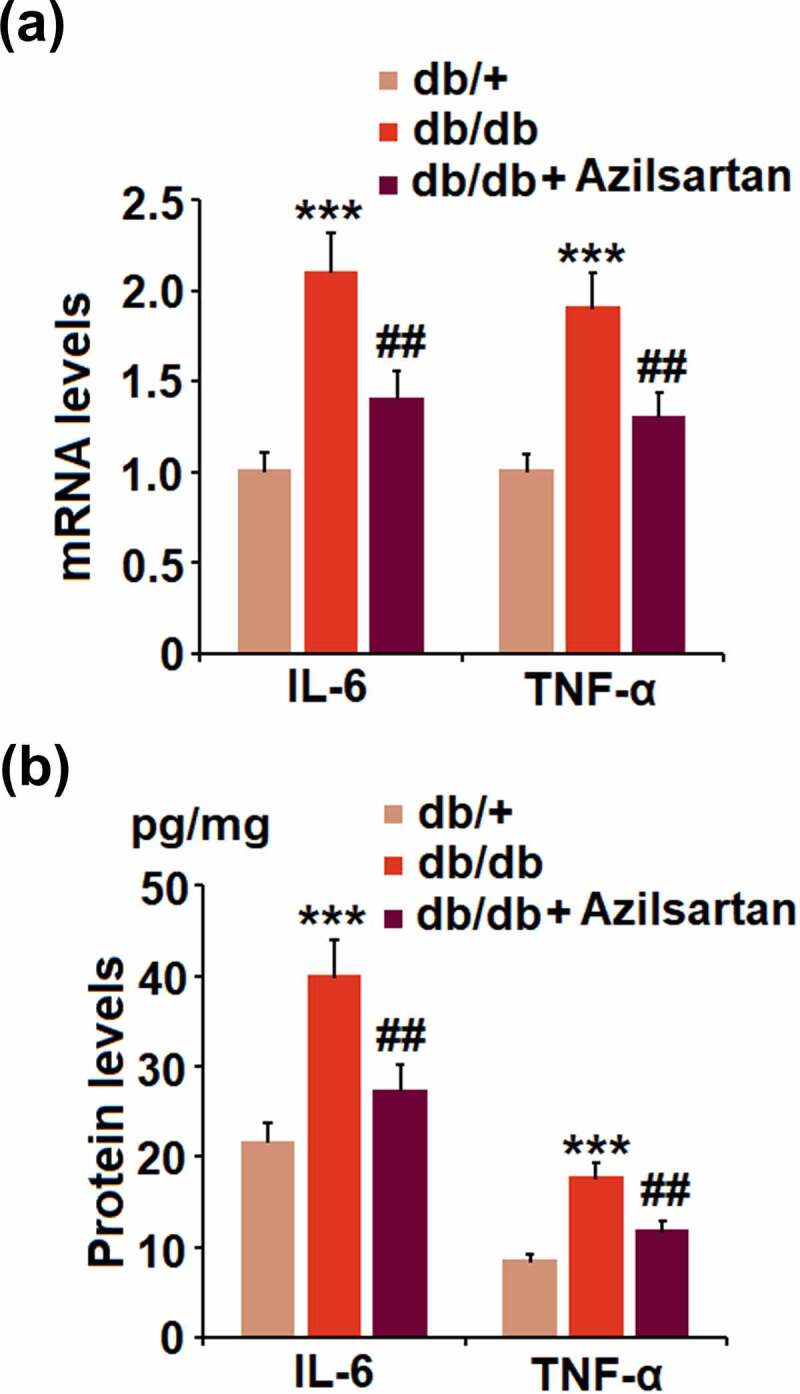
Azilsartan suppressed the expressions of the pro-inflammatory cytokines in brain vessels of diabetic mice. (A). mRNA of IL-6 and TNF-α; (B). Protein of IL-6 and TNF-α (***, p < 0.005 vs. db/+ group; ##, P < 0.01 vs. db/db group)

We further investigated the profiles of inflammation in the isolated brain vessels from animal models. We found that the elevated expression levels of IL-6 and TNF-α in T2DM mice were pronouncedly inhibited by treatment with Azilsartan (Figure 3a). In addition, as illustrated in Figure 3b, compared to the control, the release of IL-6 in brain vessels of T2DM mice was significantly elevated from 21.5 pg/mg protein to 39.8 pg/mg protein and greatly suppressed to 27.2 pg/mg protein by the administration of Azilsartan. The concentrations of TNF-α in brain vessels of normal mice, T2DM mice, and Azilsartan treated T2DM mice were 8.3, 17.5, 11.6 pg/mg protein, respectively. These data revealed that the severe inflammation in brain vessels of T2DM mice was significantly ameliorated by Azilsartan.

## Azilsartan inhibited the expressions of KLF2 in brain vessels of diabetes mice

**Figure 4.**
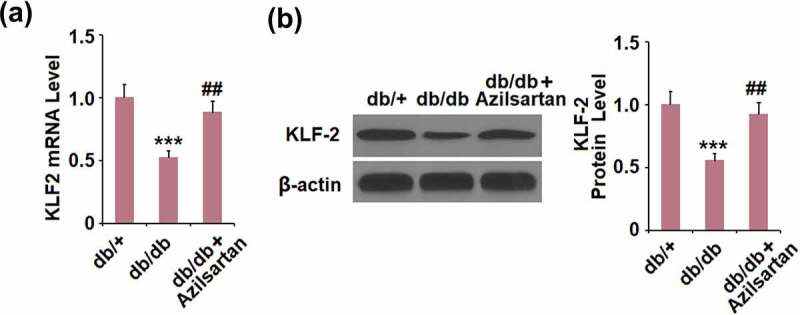
Azilsartan inhibited the expressions of KLF2 in brain vessels of diabetic mice. (A). mRNA of KLF2; (B). Protein of KLF-2 (***, p < 0.005 vs. db/+ group; ##, P < 0.01 vs. db/db group)

To further explore the mechanism underlying the regulatory effect of Azilsartan on the tight junction protein occludin, the expression level of KLF2 in brain vessels was detected. As shown in Figure 4, compared to the control, KLF2 was significantly downregulated in brain vessels of T2DM mice and dramatically upregulated by the administration of Azilsartan.

## Effects of Azilsartan on serum fasting blood glucose levels

As shown in Supplementary Figure 1, compared to the vehicle group, serum fasting blood glucose levels in db/db mice were increased. Interestingly, administration of Azilsartan significantly reduced serum fasting glucose level.

**Azilsartan alleviated high glucose-induced endothelial monolayer permeability in bEnd.3 brain endothelial cells by increasing occludin**

**Figure 5.**
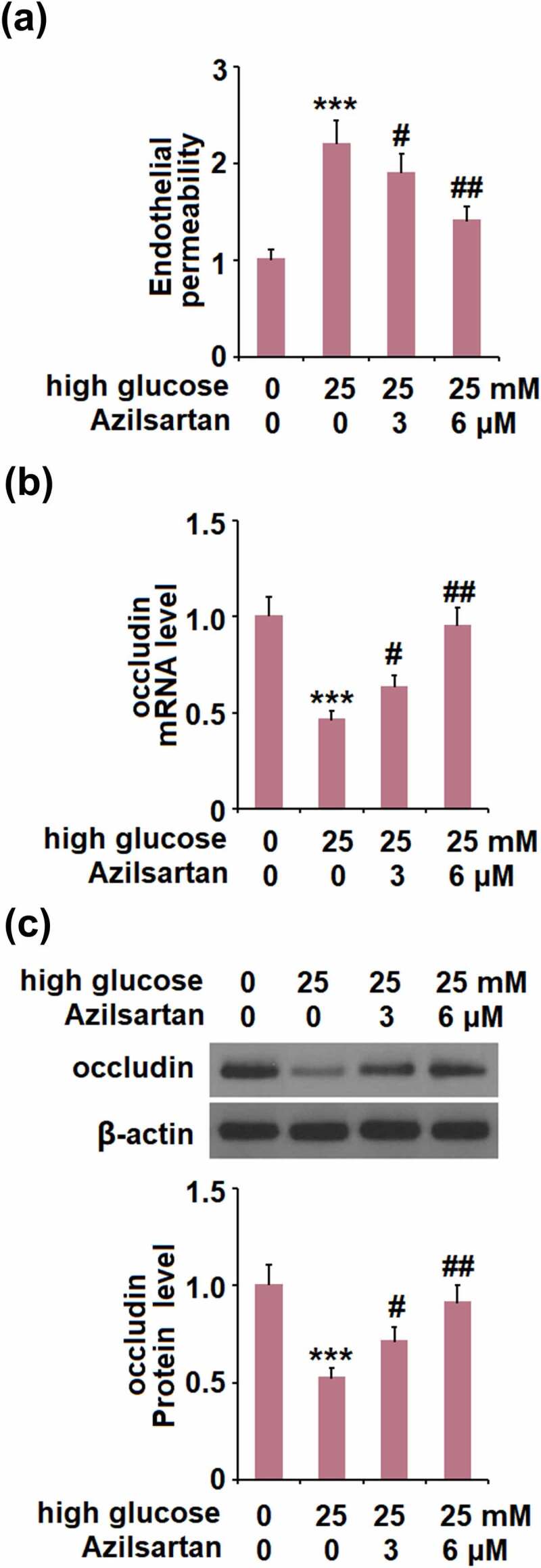
Azilsartan alleviated high glucose (25 mM)-induced endothelial monolayer permeability in bEnd.3 brain endothelial cells by increasing occludin. Cells were stimulated with high glucose (25 mM) with or without Azilsartan (3, and 6 μM) for 24 hours. (A). Endothelial permeability; (B). mRNA of occludin); (C). Protein of occludin (***, p < 0.005 vs. control group; #, ##, P < 0.05, 0.01 vs. high glucose group)

Cells were stimulated with high glucose (25 mM) with or without Azilsartan (3, and 6 μM) for 24 hours, followed by evaluating the *in-vitro* endothelial monolayer permeability. As shown in Figure 5A, compared to the control, the FITC fluorescent intensity was significantly enhanced by stimulation with high glucose, but greatly weakened by the presence of Azilsartan. In addition, the declined expression level of occludin induced by high glucose was significantly elevated by treatment with Azilsartan (Figures 5b-c). These data indicate that high glucose-induced endothelial monolayer permeability and decreased expression of occludin in bEnd.3 brain endothelial cells were significantly alleviated by Azilsartan.

**The protective effects of Azilsartan against the high glucose-induced expression of occludin and endothelial monolayer permeability are mediated by KLF2**

**Figure 6.**
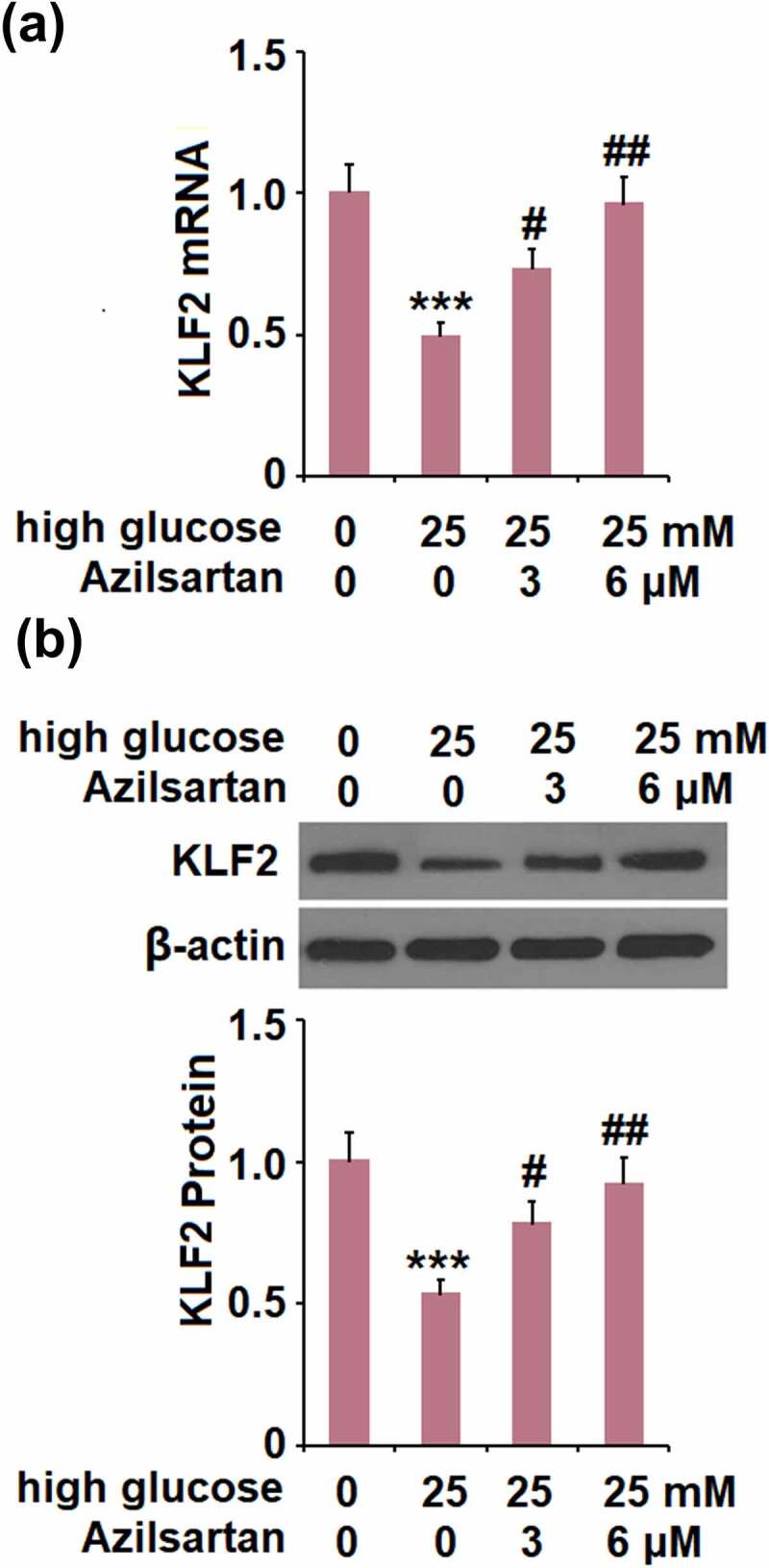
Azilsartan restored high glucose (25 mM)-induced reduction of KLF2 in bEnd.3 brain endothelial cells. Cells were stimulated with high glucose (25 mM) with or without Azilsartan (3, and 6 μM) for 24 hours. (A). mRNA of KLF2; (B). Protein of KLF2 (***, p < 0.005 vs. control group; #, ##, P < 0.05, 0.01 vs. high glucose group)

We further investigated the expression of KLF2 in treated bEnd.3 brain endothelial cells. Compared to the control group, the expression level of KLF2 was significantly suppressed by stimulation with high glucose, and greatly promoted by the introduction of Azilsartan (Figure 6).

**Figure 7.**
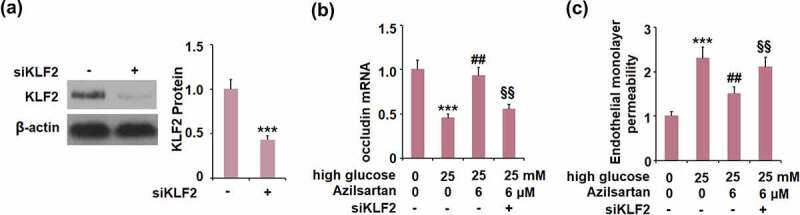
Silencing of KLF2 abolished the protective effects of Azilsartan against high glucose-induced expression of occludin and endothelial monolayer permeability in bEnd.3 brain endothelial cells. Cells were transfected with KLF2 siRNA, followed by stimulation with high glucose (25 mM) with or without Azilsartan (6 μM) for 24 hours. (A). Western blot analysis revealed successful knockdown of KLF2; (B). Expression of occludin; (C). Endothelial monolayer permeability (***, p < 0.005 vs. control group; ##, P < 0.05, 0.01 vs. high glucose group; §§, high glucose+ Azilsartan group)

To verify the mechanism underlying the regulatory effect of Azilsartan on occludin expression and monolayer permeability, cells were transfected with KLF2 siRNA, followed by stimulation with high glucose (25 mM) with or without Azilsartan (6 μM) for 24 hours. As shown in Figure 7a, compared to the control group, the expression of KLF2 was significantly suppressed in siRNA transfected cells, indicating that the KLF2 silenced bEnd.3 brain endothelial cells were successfully established. Interestingly, the declined expression of occludin in high glucose-stimulated cells was significantly elevated by treatment with Azilsartan, and further reversed by silencing KLF2 in bEnd.3 brain endothelial cells. Additionally, the enhanced FITC fluorescent intensity in high glucose-stimulated cells was dramatically weakened by the introduction of Azilsartan and pronouncedly reversed by silencing KLF2 in bEnd.3 brain endothelial cells. These data indicated that the protective effects of Azilsartan against the high glucose-induced expression of occludin and endothelial monolayer permeability in bEnd.3 brain endothelial cells were significantly abolished by the silence of KLF2.

## Azilsartan reduced expressions of NOX-2 and NOX-4 in HG-challenged bEnd.3 brain endothelial cells

To explore the effects of Azilsartan on oxidative stress in HG-stimulated bEnd.3 brain endothelial cells, the expression of oxidative stress-related markers NADPH oxidase NOX-2 and NOX-4 were measured. As expected, mRNA levels of NOX-2 and NOX-4 were remarkably upregulated in the HG-treated group, whereas Azilsartan downregulated their levels, relative to the HG group (Supplementary Figure 2).

## Discussion

Persistent hyperglycemia is one of the damage factors for the pathological changes of the BBB and the destruction of regional integrity in DM, which contributes to basal membrane thickening of the BBB, calcium deposition of the microvessel wall, decreased cortical capillary density, and the impaired BBB barrier function [19]. In addition, hyperglycemia induces damages to the endothelium by modifying low-density lipoprotein (LDL), triggering oxidative stress, and destroying the function of endothelial cells [20]. The BBB is located between the blood and nervous tissues and prevents the substances from entering the brain tissues. It is a cell complex composed mainly of capillary endothelial cells, tight junctions, basal membrane, pericytes, and astrocytes [21]. Tight junction proteins, such as occludin, form the structural basis for the integrity of the BBB. Under pathological conditions, such as inflammation, ischemia, and infection, the downregulation of occludin in the endothelial cells in brain microvessels is closely related to the increased BBB permeability [22]. In the present study, the permeability of BBB was found to be significantly increased in diabetic mice, which was an effect also observed in the endothelial monolayer constructed by the high glucose-challenged brain vascular endothelial cells. In addition, the increased permeability of the BBB or the brain vascular endothelial monolayer was accompanied by a decrease in the expression of occludin. These data indicate that the stimulation of hyperglycemia may damage the structure of the BBB, which is consistent with the previously described reports [23,24]. Through Azilsartan treatment, we found that the enhanced BBB or endothelial monolayer permeability and the downregulated occludin were significantly reversed, indicating a potential protective effect of Azilsartan against BBB disruption induced by hyperglycemia.

DM is also regarded as a kind of inflammatory disease, which involves multiple inflammatory factors, such as IL-6, C-reactive protein, plasminogen activator inhibitor-1, tumor necrosis factor-α (TNF-α), and adiponectin [25]. TNF-α induces an increase in the BBB permeability by directly acting on endothelial cells or indirectly promoting the production of IL-6 and IL-1β [26,27]. IL-6 changes BBB permeability by decreasing the tissue compactness and downregulating tight junction proteins [28]. In the present study, we found that the production of inflammatory factors was significantly increased in the brain vessels of diabetic mice and dramatically alleviated by treatment with Azilsartan, indicating a protective effect of Azilsartan against hyperglycemia-induced inflammation in the brain. In our future work, more elements will be taken into consideration that are responsible for the protective effect of Azilsartan on hyperglycemia-induced damages, such as oxidative stress, which is also believed to be an important mediator for the disruption of the BBB structure and severe inflammation induced by hyperglycemia [29,30].

Kruppel-like factor 2 (KLF2) is an important member of the family of Kruppel transcriptional factors and is involved in the process of cellular differentiation and tissue development [31]. Recently, it has been reported that KLF2 exerts anti-inflammatory properties, stabling vascular endothelium, and preventing thrombogenesis [32]. The animal experiments revealed that the expression of occludin could be regulated by KLF2. After brain injury, inducing the expression of KLF2 exerts a significant brain protective property by regulating the BBB permeability [33]. In the present study, the expression of KLF2 was significantly downregulated both in diabetic mice and high glucose-treated endothelial cells and reversed by treatment with Azilsartan. In addition, the protective effects of Azilsartan against the high glucose-induced expressions of occludin and endothelial monolayer permeability in bEnd.3 brain endothelial cells were dramatically abolished by silencing KLF2, indicating that KLF2 might be an important mediator in the protective function of Azilsartan. In our future work, the underlying mechanism will be further confirmed in the animal experiments, such as co-administration with the KLF2 inhibitor.

There are several limitations to the current study. Firstly, we used leptin receptor-deficient (db/db) mice in this study. However, the T2DM-like manifestations in db/db mice are secondary to genetic mutations, which do not reflect the real disease etiology in their human counterparts [34]. Secondly, the corrections of T2DM and hypertension still need to be elucidated. It has been reported that inhibition of endogenous angiotensin II receptors with losartan resulted in a greater decrease in blood pressure in leptin receptor-mutated rodents than in lean rats [35], suggesting that db/db mice might have increased sensitivity to angiotensin-II. Therefore, it might be difficult to compare the efficacy of Azilsartan in the db/db mice and human T2DM patients. Thirdly, although using a cell model stimulated with high glucose, we demonstrated that the effects of Azilsartan in reducing endothelial monolayer permeability and increasing occludin against high glucose are mediated by KLF2. It should be noted that *in vitro* and *in vivo* conditions are very different. Future investigations of KLF2-deficient mice will help confirm the involvement of KLF2.

## Conclusion

In summary, our data show that Azilsartan prevents hyperglycemia-induced hyperpermeability of the BBB by activating the KLF2/occludin signaling pathway. These findings shed a light on the therapeutic potential of Azilsartan in T2DM- associated neurological disorders.

## Supplementary Material

Supplemental Material

## References

[1] Juan J, Yang H. Prevalence, Prevention, and Lifestyle Intervention of Gestational Diabetes Mellitus in China. Int J Environ Res Public Health. 2020;17(24):9517. 10.3390/ijerph17249517 PMC7766930 33353136

[2] Tumminia A, Vinciguerra F, Parisi M, et al. Type 2 Diabetes Mellitus and Alzheimer’s Disease: Role of Insulin Signalling and Therapeutic Implications. Int J Mol Sci. 2018 Oct 24;19(11):3306. 10.3390/ijms19113306 PMC6275025 30355995

[3] Umegaki H, Hayashi T, Nomura H, et al. Cognitive dysfunction: an emerging concept of a new diabetic complication in the elderly. Geriatr Gerontol Int. 2013;13(1):28–34. DOI:10.1111/j.1447-0594.2012.00922.x. 22882533

[4] Cukierman-Yaffee T. The relationship between dysglycemia and cognitive dysfunction. Curr Opin Invest Drugs. 2009;10:70–74. 19127489

[5] Biessels GJ. Sweet memories: 20 years of progress in research on cognitive functioning in diabetes. Eur J Pharmacol. 2013;719(1–3):153–160. 23872409 10.1016/j.ejphar.2013.04.055

[6] Prasad S, Sajja RK, Naik P, et al. Diabetes mellitus and blood-brain barrier dysfunction: an overview. J Pharmacovigil. 2014;2:125. 25632404 10.4172/2329-6887.1000125 PMC4306190

[7] Daneman R, Prat A. The blood-brain barrier.Cold Spring Harb Perspect Biol. 2015;7:a020412. 25561720 10.1101/cshperspect.a020412 PMC4292164

[8] Chehade JM, Haas MJ, Mooradian AD. Diabetes-related changes in rat cerebral occludin and zonula occludens-1 (ZO-1) expression. Neurochem Res. 2002;27(3):249–252. 11958524 10.1023/a:1014892706696

[9] VanGilder RL, Kelly KA, Chua MD, et al. Administration of sesamol improved blood-brain barrier function in streptozotocin-induced diabetic rats. Exp Brain Res. 2009;197(1):23–34. 19565232 10.1007/s00221-009-1866-6

[10] Kook SY, Seok Hong H, Moon M, et al. Disruption of blood-brain barrier in Alzheimer disease pathogenesis. Tissue Barriers. 2013;1:e23993. DOI:10.4161/tisb.23993 24665385 PMC3887048

[11] Chiplunkar AR, Curtis BC, Eades GL, et al. The Kruppel-like factor 2 and Kruppel-like factor 4 genes interact to maintain endothelial integrity in mouse embryonic vasculogenesis. BMC Dev Biol. 2013;13(1):40. DOI:10.1186/1471-213X-13-40. 24261709 PMC4222490

[12] Hjermitslev M, Grimm DG, Wehland M, et al. Azilsartan medoxomil, an angiotensin ii receptor antagonist for the treatment of hypertension. Basic Clin Pharmacol Toxicol. 2017;121(4):225–233. 28444983 10.1111/bcpt.12800

[13] Pradhan A, Tiwari A, Sethi R. Azilsartan: current evidence and perspectives in management of hypertension. Int J Hypertens. 2019;2019:1824621. 31885897 10.1155/2019/1824621 PMC6925743

[14] Liu H, Mao P, Wang J, et al. Azilsartan, an angiotensin II type 1 receptor blocker, attenuates tert-butyl hydroperoxide-induced endothelial cell injury through inhibition of mitochondrial dysfunction and anti-inflammatory activity. Neurochem Int. 2016;94:48–56. 26879328 10.1016/j.neuint.2016.02.005

[15] Gupta V, Dhull DK, Joshi J, et al. Neuroprotective potential of azilsartan against cerebral ischemic injury: possible involvement of mitochondrial mechanisms. Neurochem Int. 2020;132:104604. 31751621 10.1016/j.neuint.2019.104604

[16] Nong A, Li QF, Huang ZJ, et al. MicroRNA miR-126 attenuates brain injury in septic rats via NF-κB signaling pathway. Bioengineered. 2021;12(1):2639–2648. 34115555 10.1080/21655979.2021.1937905 PMC8806573

[17] Chen LQ, Zhu QL, Lu LW, et al. MiR-132 inhibits migration and invasion and increases chemosensitivity of cisplatin-resistant oral squamous cell carcinoma cells via targeting TGF-β1. Bioengineered. 2020;11(1):91–102. 31906769 10.1080/21655979.2019.1710925 PMC6961592

[18] Liu WY, Miao YQ, Zhang L, et al. MiR-211 protects cerebral ischemia/reperfusion injury by inhibiting cell apoptosis. Bioengineered. 2020;11(1):189–200. 32050841 10.1080/21655979.2020.1729322 PMC7039642

[19] Yamagishi S, Imaizumi T. Diabetic vascular complications: pathophysiology, biochemical basis and potential therapeutic strategy. Curr Pharm Des. 2005;11(18):2279–2299. 16022668 10.2174/1381612054367300

[20] Calderon-Garciduenas L, Villarreal-Calderon R, Valencia-Salazar G, et al. Systemic inflammation, endothelial dysfunction, and activation in clinically healthy children exposed to air pollutants. Inhal Toxicol. 2008;20(5):499–506. DOI:10.1080/08958370701864797. 18368620

[21] Stauber WT, Ong SH, McCuskey RS. Selective extravascular escape of albumin into the cerebral cortex of the diabetic rat. Diabetes. 1981;30(6):500–503. 7014313 10.2337/diab.30.6.500

[22] Kumar TP, Antony S, Gireesh G, et al. Curcumin modulates dopaminergic receptor, CREB and phospholipase C gene expression in the cerebral cortex and cerebellum of streptozotocin induced diabetic rats. J Biomed Sci. 2010;17(1):43. 20513244 10.1186/1423-0127-17-43 PMC2890658

[23] Zhao Z, Hu J, Gao X, et al. Hyperglycemia via activation of thromboxane A2 receptor impairs the integrity and function of blood-brain barrier in microvascular endothelial cells. Oncotarget. 2017;8(18):30030–30038. DOI:10.18632/oncotarget.16273. 28415790 PMC5444723

[24] Rom S, Zuluaga-Ramirez V, Gajghate S, et al. Hyperglycemia-driven neuroinflammation compromises bbb leading to memory loss in both diabetes mellitus (DM) Type 1 and Type 2 mouse models. Mol Neurobiol. 2019;56(3):1883–1896. DOI:10.1007/s12035-018-1195-5. 29974394 PMC6320739

[25] Muriach M, Flores-Bellver M, Romero FJ, et al. Diabetes and the brain: oxidative stress, inflammation, and autophagy. Oxid Med Cell Longev. 2014;102158. DOI:10.1155/2014/102158 25215171 PMC4158559

[26] Deli MA, Descamps L, Dehouck MP, et al. Exposure of tumor necrosis factor-alpha to luminal membrane of bovine brain capillary endothelial cells cocultured with astrocytes induces a delayed increase of permeability and cytoplasmic stress fiber formation of actin. J Neurosci Res. 1995;41(6):717–726. DOI:10.1002/jnr.490410602. 7500373

[27] Didier N, Romero IA, Creminon C, et al. Secretion of interleukin-1beta by astrocytes mediates endothelin-1 and tumour necrosis factor-alpha effects on human brain microvascular endothelial cell permeability. J Neurochem. 2003;86(1):246–254. 12807444 10.1046/j.1471-4159.2003.01829.x

[28] Engelhardt B, Sorokin L. The blood-brain and the blood-cerebrospinal fluid barriers: function and dysfunction. Semin Immunopathol. 2009;31(4):497–511. 19779720 10.1007/s00281-009-0177-0

[29] Karam BS, Chavez-Moreno A, Koh W, et al. Oxidative stress and inflammation as central mediators of atrial fibrillation in obesity and diabetes. Cardiovasc Diabetol. 2017;16(1):120. 28962617 10.1186/s12933-017-0604-9 PMC5622555

[30] Price TO, Eranki V, Banks WA, et al. Topiramate treatment protects blood-brain barrier pericytes from hyperglycemia-induced oxidative damage in diabetic mice. Endocrinology. 2012;153(1):362–372. 22109883 10.1210/en.2011-1638 PMC3249670

[31] Jha P, Das H. KLF2 in regulation of NF-kappaB-mediated immune cell function and inflammation. Int J Mol Sci. 2017;18. 10.3390/ijms18112383 PMC5713352 29125549

[32] Atkins GB, Jain MK. Role of Kruppel-like transcription factors in endothelial biology. Circ Res. 2007;100(12):1686–1695. 17585076 10.1161/01.RES.0000267856.00713.0a

[33] Shi H, Sheng B, Zhang F, et al. Kruppel-like factor 2 protects against ischemic stroke by regulating endothelial blood brain barrier function. Am J Physiol Heart Circ Physiol. 2013;304(6):H796–805. DOI:10.1152/ajpheart.00712.2012. 23335794 PMC3602774

[34] Wang B, Chandrasekera PC, Pippin JJ. Leptin- and leptin receptor-deficient rodent models: relevance for human type 2 diabetes. Curr Diabetes Rev. 2014;10(2):131–145. 24809394 10.2174/1573399810666140508121012 PMC4082168

[35] Alonso-Galicia M, Brands MW, Zappe DH, et al. Hypertension in obese Zucker rats Role of angiotensin II and adrenergic activity. Hypertension. 1996 Dec;28(6):1047–1054. 8952595 10.1161/01.hyp.28.6.1047

