# Supplementary figures and images for: Azilsartan protects against hyperglycemia-induced hyperpermeability of the blood-brain barrier
Source: Bioengineered. 2021 Jul 16;12(1):3621–33. doi: 10.1080/21655979.2021.1948950 (PMC8806574; doi:10.1080/21655979.2021.1948950)

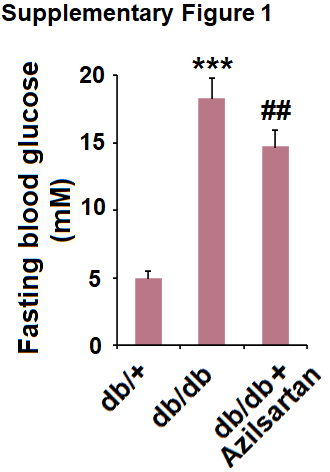

Supplement: Supplemental Material [file KBIE_A_1948950_SM6608.zip › supplementary/supplementary Figure 1.tif]

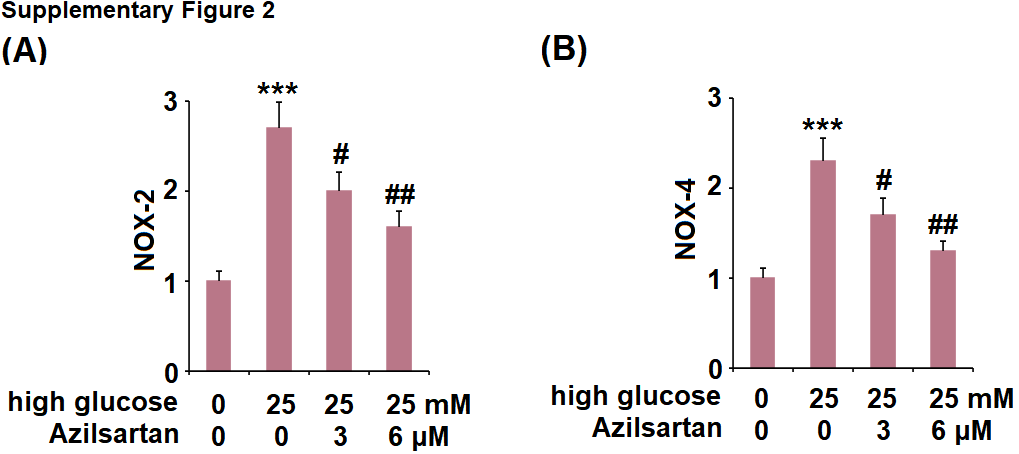

Supplement: Supplemental Material [file KBIE_A_1948950_SM6608.zip › supplementary/supplementary Figure 2.tif]
